# Functional Consequences of the *SCN5A*-p.Y1977N Mutation within the PY Ubiquitylation Motif: Discrepancy between HEK293 Cells and Transgenic Mice

**DOI:** 10.3390/ijms20205033

**Published:** 2019-10-11

**Authors:** Simona Casini, Maxime Albesa, Zizun Wang, Vincent Portero, Daniela Ross-Kaschitza, Jean-Sébastien Rougier, Gerard A. Marchal, Wendy K. Chung, Connie R. Bezzina, Hugues Abriel, Carol Ann Remme

**Affiliations:** 1 Department of Clinical and Experimental Cardiology, Heart Centre, Amsterdam UMC, Location Academic Medical Center, University of Amsterdam, Meibergdreef 9, 1105 Amsterdam, The Netherlands; s.casini@amsterdamumc.nl (S.C.); v.m.portero@amsterdamumc.nl (V.P.); g.a.marchal@amsterdamumc.nl (G.A.M.); c.r.bezzina@amsterdamumc.nl (C.R.B.); 2 Ion Channels and Channelopathies Laboratory, Institute for Biochemistry and Molecular Medicine, University of Bern, Bühlstrasse 28, 3012 Bern, Switzerland; maxime.albesa@zeiss.com (M.A.); zizun.wang@ibmm.unibe.ch (Z.W.); daniela.ross@ibmm.unibe.ch (D.R.-K.); jean-sebastien.rougier@ibmm.unibe.ch (J.-S.R.); Hugues.Abriel@ibmm.unibe.ch (H.A.); 3 Departments of Pediatrics & Medicine, Columbia University Medical Center, 1150 St Nicholas Avenue, New York, NY 10032, USA; wkc15@cumc.columbia.edu

**Keywords:** *SCN5A*, ubiquitylation, long QT syndrome, sodium current, Nedd4-2, action potential, patch-clamp, mouse model

## Abstract

Dysfunction of the cardiac sodium channel Nav1.5 (encoded by the *SCN5A* gene) is associated with arrhythmias and sudden cardiac death. *SCN5A* mutations associated with long QT syndrome type 3 (LQT3) lead to enhanced late sodium current and consequent action potential (AP) prolongation. Internalization and degradation of Na_v_1.5 is regulated by ubiquitylation, a post-translational mechanism that involves binding of the ubiquitin ligase Nedd4-2 to a proline-proline-serine-tyrosine sequence of Na_v_1.5, designated the PY-motif. We investigated the biophysical properties of the LQT3-associated *SCN5A*-p.Y1977N mutation located in the Na_v_1.5 PY-motif, both in HEK293 cells as well as in newly generated mice harboring the mouse homolog mutation *Scn5a*-p.Y1981N. We found that in HEK293 cells, the *SCN5A*-p.Y1977N mutation abolished the interaction between Na_v_1.5 and Nedd4-2, suppressed PY-motif-dependent ubiquitylation of Na_v_1.5, and consequently abrogated Nedd4-2 induced sodium current (I_Na_) decrease. Nevertheless, homozygous mice harboring the *Scn5a*-p.Y1981N mutation showed no electrophysiological alterations nor changes in AP or (late) I_Na_ properties, questioning the in vivo relevance of the PY-motif. Our findings suggest the presence of compensatory mechanisms, with additional, as yet unknown, factors likely required to reduce the “ubiquitylation reserve” of Na_v_1.5. Future identification of such modulatory factors may identify potential triggers for arrhythmias and sudden cardiac death in the setting of LQT3 mutations.

## 1. Introduction

Sodium ion influx through the α-subunit of the cardiac sodium channel (Na_v_1.5) determines the rapid upstroke of the action potential (AP), cardiomyocyte excitability and proper conduction in the heart [1]. Mutations in *SCN5A,* the gene encoding Na_v_1.5, are associated with a myriad of clinical syndromes, including Brugada syndrome (BrS), cardiac conduction disease (CCD), long QT syndrome type 3 (LQT3), atrial fibrillation, and dilated cardiomyopathy [2,3]. LQT3 is characterized by abnormal ventricular repolarization, with a prolonged QT interval on the electrocardiogram (ECG). Patients with LQT3 are at high risk for cardiac arrest due to ventricular tachyarrhythmias, in particular torsades de pointes, which usually occur during rest or sleep [1]. In LQT3, *SCN5A* mutations typically lead to an increase in late sodium current (I_NaL_), a small inward current that persists throughout the duration of the AP plateau and repolarization phase leading to AP prolongation that can in turn predispose to torsades de pointes ventricular arrhythmias and sudden cardiac death (SCD) [4,5]. Such gain of function *SCN5A* mutations often impact on the inactivation gate of the channel, thereby increasing I_NaL_ [4,5], but other mechanisms may also be involved, including altered post-translational regulation and/or degradation of Na_v_1.5-based channels. Unravelling these mechanisms may yield critical molecular insight into arrhythmogenesis and ultimately identify novel therapeutic strategies for the prevention of SCD in *SCN5A* mutation carriers.

Ubiquitylation has been shown to be implicated in the regulation of cardiac ion channels, modulating their internalization and endoplasmic reticulum-associated degradation [6]. This post-translational modification, consisting of the covalent attachment of one or several ubiquitin moieties on a lysine residue of the target protein, is a three-step enzymatic modification, ultimately resulting in an isopeptide bond being formed between ubiquitin and the target protein [7]. The cardiac sodium channel protein Na_v_1.5 possesses a proline-proline-serine-tyrosine (PPSY) sequence (designated the PY-motif) in its C-terminal cytoplasmic tail. PY-motifs are known to mediate the interaction with WW domains of ubiquitin ligases of the Nedd4 family [8,9]. Na_v_1.5 has been shown to interact with and to be a substrate of Nedd4-2 [9]. In heterologous systems, PY-motif-dependent ubiquitylation of Na_v_1.5 by Nedd4-2 leads to a decrease of sodium current (I_Na_) when the two proteins are co-expressed. Mutagenesis of the tyrosine within the PY-motif of Na_v_1.5 has been shown to abrogate the interaction between Na_v_1.5 and Nedd4-2, eliminating the functional effect of the ubiquitin ligase on the channel [9,10]. While these findings demonstrate the potential regulation of Na_v_1.5 by Nedd4-2 in vitro, the in vivo relevance of PY-motif-dependent ubiquitylation of Na_v_1.5 is unclear. Only a limited number of *SCN5A* mutations have so far been investigated in either transgenic mouse models or human induced pluripotent stem cell-derived cardiomyocytes (hiPSC-CMs), revealing both similarities and differences in mutation-induced biophysical consequences as compared to observations in expression systems (e.g., HEK293 cells) [5,11]. Hence, assessing the functional relevance of post-translational modifications such as ubiquitylation in a cardiomyocyte environment is essential.

In the present study, we identified a *SCN5A* mutation (p.Y1977N) located in the PY-motif of Na_v_1.5 in a LQT3 patient. We hypothesized that the consequent altered ubiquitylation of Na_v_1.5 would affect proper channel degradation/internalization, leading to sodium current alterations and consequent repolarization abnormalities, explaining the observed LQT3 phenotype. To investigate this hypothesis, we characterized the mutation in a heterologous expression system, and studied the in vivo relevance by generating knock-in mice harboring the murine homologous mutation *Scn5a*-p.Y1981N.

## 2. Results

### 2.1. Case Report

A female patient presented at the age of 14 complaining of shortness of breath and chest tightness. During evaluation in the emergency room she was found to have a normal echocardiogram, while a series of ECGs demonstrated QTc values ranging from 460–500 ms. During cardiac catheterization, her baseline QTc was 438–460 ms and prolonged to 509 ms upon epinephrine challenge. She previously experienced two episodes of syncope; the first when she was a toddler and riding a horse, and the second at the age of 12 when she was going down a water slide. Little is known about her family history since she was adopted. However, her father and a paternal aunt were known to have died of sudden death at the age of 31 and 16, respectively. Genetic screening for LQTS genes revealed a heterozygous missense mutation c.T5929A (p.Y1977N) in *SCN5A* located in the intracellular C-terminus of Na_v_1.5. No other rare deleterious variants were found in the other LQT genes. The patient was diagnosed with LQT3 and managed with beta-blocker treatment, restriction from competitive sports, and avoidance of QT prolonging medication. At the age of 17 she received an implantable cardioverter defibrillator (ICD). Four years later, the patient elected to have her ICD removed rather than replaced. At the age of 27 she had a cardiac arrest and was found unresponsive on the sofa and then had a seizure. Upon resuscitation, her QTc was 560 ms and the following day 600 ms. She was hospitalized and an ICD was implanted.

### 2.2. The SCN5A-p.Y1977N Mutation Abolishes the Interaction between Na_v_1.5 and Nedd4-2 and Nedd4-2-Dependent Ubiquitylation of Na_v_1.5 in HEK293 Cells

**Figure 1:**
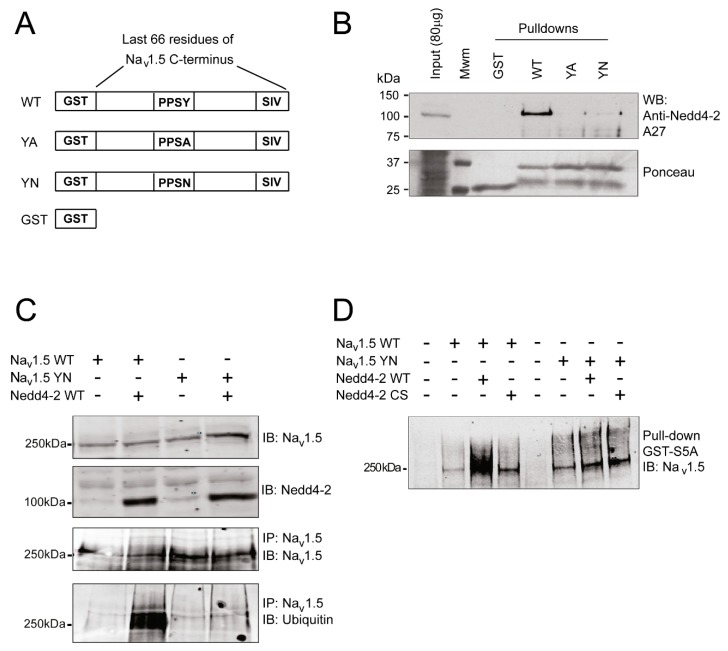
The *SCN5A*-p.Y1977N mutation abolishes the interaction between Na_v_1.5 and Nedd4-2 and Nedd4-2 ubiquitylation of Na_v_1.5. (**A**) Schematic representation of the four fusion proteins used for the pull-down experiments described in (**B**). PPSY (proline-proline-serine-tyrosine) represents the wild-type (WT) sequence of the PY-motif of Na_v_1.5, while PPSA and PPSN represent mutations of the tyrosine (Y) of the PY-motif in alanine (A) and asparagine (N), respectively. (**B**) Western blots of pull-down fractions performed on HEK293 lysates against Nedd4-2. The bottom panel shows a Ponceau staining of a representative nitrocellulose membrane, showing the presence of Glutathione S-Transferase (GST) fusion proteins for the different pull-down experiments. (**C**) Immunoprecipitation of Na_v_1.5 followed by blotting against either Na_v_1.5 or ubiquitin demonstrating ubiquitylation of the WT channel, but not of the YN channel, in the presence of Nedd4-2 WT. (**D**) Total ubiquitylated proteins from HEK293 lysates precipitated using GST-S5A fusion proteins and subsequently blotted with an antibody against Na_v_1.5, demonstrating a lack of WT Na_v_1.5 ubiquitylation in the presence of a catalytic inactive form of Nedd4-2 (Nedd4.2 CS) as well as absence of ubiquitylation of YN Na_v_1.5 in the presence of WT Nedd4-2. Data were collected from three experiments.

We previously demonstrated that mutating the essential tyrosine of the PY-motif into an alanine leads to disruption of the Na_v_1.5/Nedd4-2 interaction [9]. Here, we first assessed whether a change of this PY-motif tyrosine into asparagine, as observed in our patient, also disrupts the interaction between the two partners. To address this question, we used Glutathione S-Transferase (GST)-fusion proteins containing the last 66 amino acids of either wild-type (WT) Na_v_1.5 or with the tyrosine (Y) of the PY-motif mutated into alanine (A) or asparagine (N) (YA and YN respectively, Figure 1A). Figure 1B demonstrates that, in contrast to the WT C-terminal region, which interacts with Nedd4-2 when co-expressed in HEK293 cells, interaction of the ubiquitin ligase with either YA or YN mutant C-terminus is abolished (Figure 1B and Figure S1). We next investigated whether the disrupted interaction of YN Na_v_1.5 with Nedd4-2 abolished its Nedd4-2-dependent ubiquitylation. For this, we immunoprecipitated Na_v_1.5 from HEK293 cell lysates transiently expressing Na_v_1.5 WT or YN, with or without Nedd4-2 WT, and immunoblotted the eluate fraction with antibodies against Na_v_1.5 and ubiquitin (Figure 1C and Figure S2). In contrast to the WT channel, in presence of Nedd4-2 WT, no ubiquitylation of the YN mutant channel was observed following its co-immunoprecipitation (Figure 1C and Figure S2). These findings were corroborated by pull-down experiments performed using GST-fusion protein in which the fused peptide, which recognizes polyubiquitylated proteins, corresponds to S5A [12]. As shown in Figure 1D, the ubiquitylation level of Na_v_1.5 WT detected by this technique was found to be increased in presence of Nedd4-2 WT but not with a catalytic inactive form of Nedd4-2 (Nedd4-2 CS) suggesting that the Nedd4-2-dependent ubiquitylation of Na_v_1.5 WT is supported by the catalytic domain HECT of the ubiquitin ligase (Figure 1D and Figure S3). However, in presence of Nedd4-2 WT, the enhancement of ubiquitylation of the YN mutant channel was not observed (Figure 1D and Figure S3).

### 2.3. Nedd4-2-Dependent Regulation of Sodium Current is Abrogated by the SCN5A-p.Y1977N Mutation in HEK293 Cells

**Figure 2:**
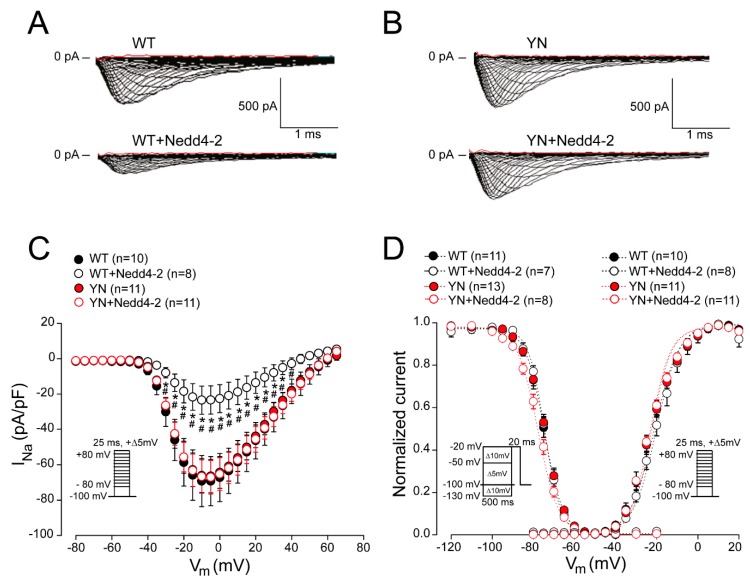
Nedd4-2-dependent regulation of sodium current is abrogated by the *SCN5A*-p.Y1977N mutation in HEK293 cells. Typical example of sodium current recordings obtained from HEK293 cells transiently transfected with *SCN5A* wild-type (WT) (**A**) or *SCN5A*-p.Y1977N (YN) (**B**) with or without Nedd4-2. Average sodium current-voltage relationship (**C**) and voltage dependence of activation and inactivation (**D**) for HEK293 cells transiently transfected with *SCN5A* WT or YN, in the presence and absence of Nedd4-2. n indicates the number of cells. Insets: voltage clamp protocols. * *p* < 0.05 vs. WT, ^#^ *p* < 0.05 vs. YN + Nedd4-2; two-way repeated measures ANOVA followed by Holm–Sidak test for post-hoc analysis.

To functionally assess whether the negative regulation of Nedd4-2 on Na_v_1.5 is abrogated by the *SCN5A*-p.Y1977N (YN)mutation, we transiently transfected HEK293 cells with *SCN5A* WT or YN in the presence or absence of Nedd4-2, and performed whole-cell patch-clamp measurements. Peak I_Na_ elicited by WT channels was significantly decreased when co-expressed with Nedd4-2 (Figure 2A,C and Table S1). In contrast, no effect of Nedd4-2 was observed on I_Na_ generated by mutant YN channels (Figure 2B,C and Table S1). Nedd4-2 did not affect voltage dependence of activation and inactivation of either WT or YN channels, as indicated by unaltered half-maximal voltage (V_1/2_) of (in)activation and slope factor *k* (Figure 2D, Table S1). Interestingly, the down-regulation of WT I_Na_ density mediated by Nedd4-2 was partly abrogated in the presence of increasing ratios of the Na_v_1.5 accessory subunit *SCN1B*. When the latter was co-transfected at a ratio of 5:1 with *SCN5A* WT, Nedd4-2 caused a less pronounced reduction in I_Na_ density as compared to co-transfection at 1:1 ratio (Figure S6, Table S2). These changes were not accompanied by differences in mRNA expression levels of *SCN5A* and *NEDD4L* (Nedd4-2) (Figure S7), indicating that the observed effects were only due to different *SCN1B* expression levels.

### 2.4. Cell Surface Expression of Na_v_1.5 in HEK293 Cells is Altered by the SCN5A-p.Y1977N Mutation

**Figure 3:**
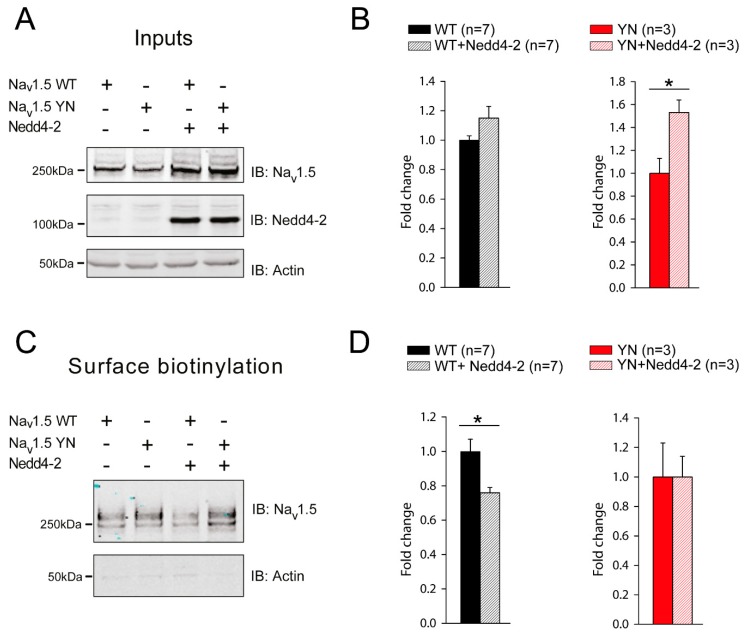
The *SCN5A*-p.Y1977N mutation abolishes the Nedd4-2 mediated decrease of Na_v_1.5 cell surface expression. (**A**) HEK293 cells were transiently transfected with the indicated plasmids and whole cell lysates were blotted against Na_v_1.5, Nedd4-2 or actin. (**B**) Fluorescence intensity of Na_v_1.5 wild-type (WT) and Y1977N (YN) with or without Nedd4-2, was quantified with LICOR Odyssey software. Data are expressed as fold change compared to the WT or YN group. (* *p* < 0.05, Unpaired Student’s *t*-test; n corresponds to the number of experiments performed). (**C**) Western blot of a representative cell surface biotinylation assay in HEK293 cells transiently expressing Na_v_1.5 WT or YN with or without Nedd4-2. (**D**) Quantification of Na_v_1.5 protein at the cell surface in the different conditions described in panel C using LICOR Odyssey software. Data are expressed as fold change compared to the WT or YN group. (* *p* < 0.05, Unpaired Student’s *t*-test; *n* corresponds to the number of experiments performed).

Nedd4-2 dependent decrease of I_Na_ in HEK293 cells has previously been shown to be the consequence of reduced cell surface expression of Na_v_1.5 due (at least in part) to an increased internalization of channels [10]. We therefore investigated in greater detail the effects of the YN mutation on Nedd4-2 dependent Na_v_1.5 cell surface expression. Overexpression of Nedd4-2 had no significant effect on the total expression of WT Na_v_1.5 in HEK293 cells, while it significantly increased the total protein level of YN Na_v_1.5 (Figure 3A,B and Figure S4). Using cell surface biotinylation assays, we next investigated cell surface expression of WT and YN Na_v_1.5 in the presence and absence of Nedd4-2. Figure 3C,D show that, while WT Na_v_1.5 is significantly decreased at the cell surface upon Nedd4-2 co-transfection (consistent with the observed Nedd4-2 dependent decrease in I_Na_), cell membrane expression of YN Na_v_1.5 is not altered by the ubiquitin ligase (Figure 3C,D and Figure S5).

### 2.5. Scn5a-p.Y1981N Mice Show Unaltered Cardiac Electrical Properties In Vivo and Ex Vivo

**Figure 4:**
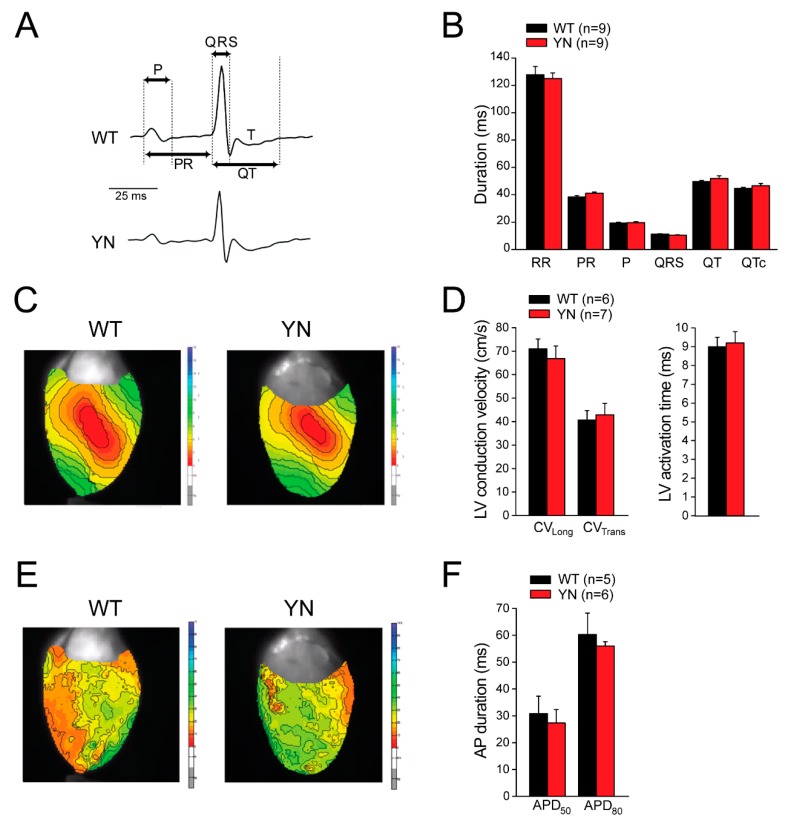
Surface ECG measuraments and optical mapping in isolated Langendorff-perfused mice hearts. (**A**) Typical surface ECG recordings in wild-type (WT) and *Scn5a*-p.Y1981N (YN) mice. Arrows indicate how the various ECG intevals were measured. RR intervals were defined as the interval in ms between two consecutive R waves. (**B**) Average data for ECG parameters. No differences were observed between WT and YN mice (*n* indicates number of mice). (**C**) Typical left ventricular (LV) activation maps from an isolated WT and YN mouse heart paced at a cycle length of 120 ms.(**D**) Average LV longitudinal (CV_long_) and LV transversal (CV_trans_) conduction velocity and LV activation time in WT and YN hearts. (**E**) Typical examples of LV activation maps from an isolated WT and YN mouse heart during stimulation at a cycle length of 150 ms. (**F**) Average values for action potential duration (APD) at 50% and 80% repolarization (APD_50_ and APD_80_) in WT and YN hearts. *n* indicates number of mice.

To assess the in vivo relevance of the PY-motif-dependent ubiquitylation of Na_v_1.5, we generated a knock-in mouse line harboring the *Scn5a*-p.Y1981N mutation, the murine homolog of the human *SCN5A*-p.Y1977N mutation. Heterozygous and homozygous *Scn5a*-p.Y1981N (YN) mice were born at a Mendelian frequency, were viable and fertile, and displayed no physical or anatomical abnormalities (including body and heart weight), and hence only homozygous YN mice were further investigated in detail. Surface ECG measurements in anaesthetized mice showed no differences in ECG parameters between WT and YN mice (Figure 4A,B). Moreover, similar beating rates were observed in both groups (486 ± 22.1 bpm in WT mice and 484.1 ± 15.4 bpm in YN mice).

In line with the in vivo ECG results, ex vivo optical mapping experiments revealed similar left ventricular activation times and unaltered longitudinal and transversal conduction velocities in isolated WT and YN Langendorff-perfused hearts (Figure 4C,D). Ventricular repolarization was also unaffected by the YN mutation, as indicated by similar values of AP duration (APD) at 50% and 80% repolarization (APD_50_ and APD_80_) in WT and YN isolated hearts (Figure 4E,F).

### 2.6. Action Potential and (Late) Sodium Current Characteristics Are Unaltered in Isolated Scn5a-p.Y1981N Cardiomyocytes

**Figure 5:**
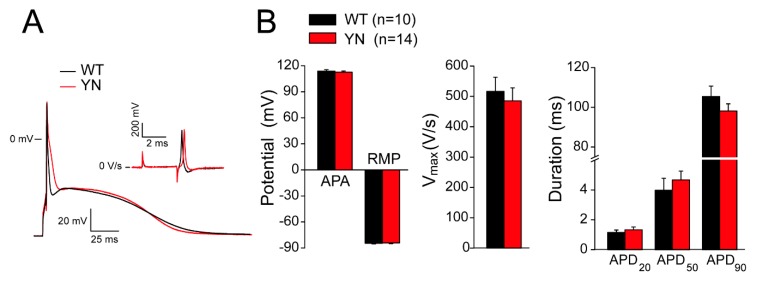
Action potential properties in isolated wild-type and *Scn5a*-p.Y1981N mouse cardiomyocytes. (**A**) Typical examples of action potentials (APs) triggered at 2 Hz and first derivative (dV/dt) of the AP upstroke (inset) in wild-type (WT) and *Scn5a*-p.Y1981N (YN) mouse cardiomyocytes. (**B**) Average values for AP amplitude (APA), resting membrane potential (RMP), maximal upstroke velocity (V_max_; measured as the maximal dV/dt of the AP upstroke) and AP duration at 20%, 50%, and 90% repolarization (APD_20_, APD_50_, APD_90_) at the stimulation frequency of 2 Hz in WT (*n* = 10 cardiomyocytes from six mice) and YN cardiomyocytes (*n* = 14 from five mice).

The consequences of the *Scn5a*-p.Y1981N mutation on cellular electrophysiological properties was next investigated by patch-clamp analysis in left ventricular cardiomyocytes isolated from WT and homozygous YN mice. APs were recorded using the perforated-patch technique at stimulation frequencies of 2 and 4 Hz, as shown in Figure 5A and Figure S8A, respectively. In accordance with the optical mapping experiments, maximal upstroke velocities (V_max_; calculated as the maximal first derivative of the AP upstroke) and AP durations at 20%, 50% and 90% repolarization (APD_20_, APD_50_, APD_90_) were similar in WT and YN cardiomyocytes at both frequencies of stimulations (Figure 5B and Figure S8B, Table S3). Other parameters, such as AP amplitude (APA) and resting membrane potential (RMP), also did not differ between the two groups (Figure 5B and Figure S8B, Table S3).

**Figure 6:**
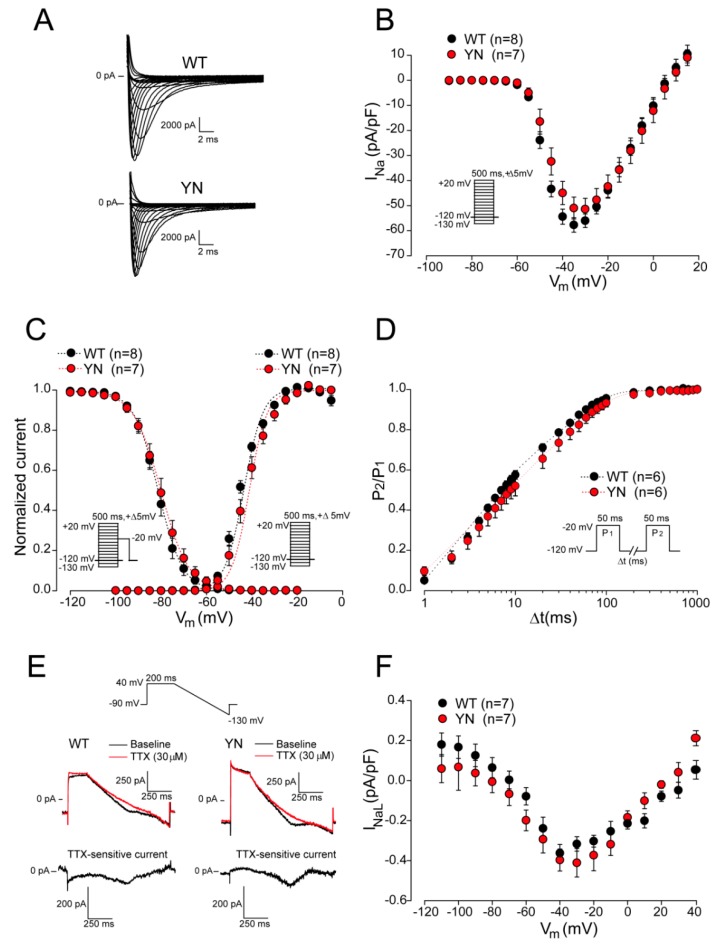
Sodium current properties in wild-type and *Scn5a*-p.Y1981N cardiomyocytes. (**A**) Representative example of sodium current (I_Na_) traces recorded in wild-type (WT) and in *Scn5a*-p.Y1981N (YN) cardiomyocytes during a 5-mV ascending voltage step protocol. (**B**) Average I_Na_-voltage relationship (*n* = 8 cardiomyocytes from six WT mice and *n* = 7 cardiomyocytes from four YN mice), (**C**) voltage dependence of activation and inactivation (*n* = 8 cardiomyocytes from six WT mice and *n* = 7 from four YN mice) and (**D**) time course of recovery from inactivation (*n* = 6 cardiomyocytes from four WT mice and *n* = 6 cardiomyocytes from four YN mice) for WT and YN cardiomyocytes. Insets: voltage clamp protocols. (**E**) Typical late sodium current (I_NaL_) traces recorded using a descending ramp protocol (upper panel) in WT and YN cardiomyocytes before (baseline) and after application of 30 µM tetrodotoxin (TTX). TTX-sensitive currents were obtained by subtraction of the current measured in the presence of TTX from the current measured earlier in the absence of TTX. (**F**) Average I_NaL_-voltage relationship for WT (*n* = 7 cardiomyocytes from four mice) and YN cardiomyocytes (*n* = 6 from five mice).

Figure 6A shows representative examples of peak I_Na_ evoked by depolarizing steps from a holding potential of –120 mV for WT and YN cardiomyocytes. No changes in average I_Na_ density were observed between WT and YN cardiomyocytes (Figure 6B, Table S4). Voltage dependence of (in)activation parameters (V_1/2_ of (in)activation and *k*) were also similar in both groups (Figure 6C, Table S4). The time course of recovery from inactivation was assessed with a two-pulse protocol as depicted in Figure 6D. Fast and slow time constants (τ_f_ and τ_s_) of recovery from inactivation were not different between WT and YN cardiomyocytes (Figure 6D, Table S4). Since LQT3 is typically associated with an enhanced I_NaL_, we performed I_NaL_ measurements in WT and YN cardiomyocytes using a descending ramp after a 200-ms prepulse to 40 mV (Figure 6E). The advantage of using a ramp protocol instead of a single step protocol, is that the ramp protocol allows measurements of I_NaL_ across a dynamic voltage range simulating a plateau and repolarization phase of an AP [5]. I_NaL_ was measured as tetrodotoxin (TTX)-sensitive current obtained by subtraction of the current recorded in the presence of 30 µM TTX from the current recorded earlier in the absence of TTX (Figure 6E). The average I_NaL_-voltage relationships shown in Figure 6F demonstrate that I_NaL_ was not increased in YN cardiomyocytes as compared to WT. Taken together, these findings reveal a lack of electrophysiological alterations in homozygous *Scn5a*-p.Y1981N mice.

## 3. Discussion

We here report a case of a female patient with a history of recurrent syncopal episodes, cardiac arrest and QT interval prolongation. Genetic analysis identified a heterozygous *SCN5A*-p.Y1977N mutation in the PY-motif of Na_v_1.5, leading to a diagnosis of LQT3. The same mutation and patient was previously reported in a large cohort of Long QT syndrome cases [13]. Several studies have shown the relevance of the PY-motif-dependent ubiquitylation of the cardiac sodium channel in vitro [6,9,10], but the in vivo role of this process has been unclear. We hypothesized that defective ubiquitylation of Na_v_1.5 due to the mutation within its PY-motif would lead to accumulation of the channel at the cell surface, resulting in electrophysiological alterations. To investigate our hypothesis, we conducted experiments in both a heterologous expression system and a homozygous mouse line harboring the *Scn5a*-p.Y1981N mutation, the murine homologue of human *SCN5A*-p.Y1977N. In HEK293 cells, the *SCN5A*-p.Y1977N mutation abolished the interaction between Na_v_1.5 and the ubiquitin ligase Nedd4-2, suppressed PY-motif-dependent ubiquitylation of Na_v_1.5, and consequently abrogated Nedd4-2 induced I_Na_ decrease. Nevertheless, homozygous mice harboring the *Scn5a*-p.Y1981N mutation showed no electrophysiological alterations nor changes in AP or (late) sodium current properties, questioning the in vivo relevance of the PY-motif.

### 3.1. PY-motif-Dependent Ubiquitylation of Na_v_1.5: In Vitro Evidence

Ubiquitylation of membrane proteins, including ion channels, is functionally linked to their internalization and degradation via lysosomal and proteasomal pathways [14]. During this process, ubiquitin attaches to lysine groups of membrane proteins through the activity of a ubiquitin-activating enzyme (E1), a ubiquitin-conjugating enzyme (E2), and a ubiquitin ligase (E3). For ion channels, ubiquitin ligases of the Nedd4 family (including, amongst others, Nedd4-1, Nedd4-2 and WWP2) appear to be predominantly involved in the ubiquitylation process, which typically bind to a PY-motif (designated by the sequence(P/L)PxY within the channel protein [6,14]. The latter has been confirmed in several in vitro studies demonstrating the loss of interaction between Nedd4 ubiquitin ligases and ion channels such as Na_v_1.5 and KCNQ1 upon modification of the PY-motif [9,10,15,16,17]. In our previous study, we mutated the seven amino acids PPSYDSV of Na_v_1.5 (forming the extended PY-motif of the channel) into alanine and observed an essential role for the tyrosine in the interaction between Na_v_1.5 and the WW domain of Nedd4 ligases [10]. In the current study, we show that mutating the tyrosine into asparagine has similar effects, i.e., abolition of the Na_v_1.5-Nedd4-2 interaction in HEK293 cells, confirming the crucial role of this amino acid in the formation of a complex with WW domains. Functionally, Nedd4-2 has been shown to increase Na_v_1.5 internalization rate at the cell surface in HEK293 cells [10]. Nedd4-2 dependent internalization of the epithelial sodium channel ENaC was also impaired secondary to a mutation in its PY-motif [18]. Similarly, we observed that the reduction in wild-type Na_v_1.5 cell surface expression induced by Nedd4-2 was abrogated by the *SCN5A*-p.Y1977N mutation, suggesting a PY-motif-dependent internalization of Na_v_1.5 by Nedd4-2. The fate of Na_v_1.5 channels after their internalization by Nedd4-2 remains unclear since overexpression of Nedd4-2 had no significant effect on the total expression of wild-type Na_v_1.5 in HEK293 cells, making channel degradation less likely. This observation is in contradiction with results obtained for the epithelial sodium channel ENaC, which have been shown to be either recycled at the cell membrane or degraded by the lysosome or the proteasome pathways after their internalization by Nedd4-2 [19]. Hence, we can hypothesize that Na_v_1.5 channels are similarly stored in an unknown intracellular compartment following Nedd4-2 induced internalization, waiting to be recycled at the cell surface.

### 3.2. In Vivo Relevance of PY-motif-Dependent Ubiquitylation

The vast majority of studies investigating the role of ubiquitylation of ion channels have been performed in heterologous expression systems, and only very few studies have explored the functional relevance of the PY-motif in vivo. Mutations in PY-motifs of subunits constituting the epithelial sodium channel ENaC have been associated with Liddle syndrome, an inherited form of hypertension caused by increased expression of ENaC at the cell membrane and consequent enhanced channel activity [20,21]. The functional relevance of Nedd4-2 in this process was suggested by the observation that kidney-specific deletion of Nedd4-2 led to salt-sensitive hypertensions [22]. Similarly, nociceptive neuron-specific knock-out of Nedd4-2 resulted in dysregulation and altered expression of neuronal sodium channels Na_v_1.7 and Na_v_1.8, accompanied by an altered nociceptive pain phenotype [17]. These examples underline the potential in vivo relevance of PY-motif and Nedd4-2 dependent ubiquitylation. However, while ubiquitylation of Na_v_1.5 is detected in mouse heart [9], very few studies have investigated its functional relevance in the cardiomyocyte environment. Recent findings in HEK293 cells and neonatal rat cardiomyocytes indicate that increased calcium levels (as observed during heart failure) enhance Nedd4-2 expression and consequent ubiquitylation and downregulation of Na_v_1.5 [23]. Furthermore, mice deficient for the Nedd4-2 C2 isoform (isoform containing a C2 domain in the N-terminus) displayed electrocardiographic signs of cardiac conduction and repolarization abnormalities in the absence of overt cardiac structural abnormalities [24]. However, no molecular or functional measurements of ion channel expression/function were performed, and it is unclear to what extent the observed alterations are primary or secondary to the renal phenotype present in these mice [24]. Our current study is the first to directly investigate the functional in vivo role of the PY-motif in the cardiomyocyte environment. We hypothesized that a decreased or defective ubiquitylation of Na_v_1.5 due to the PY-motif mutation would lead to accumulation of the channel at the cell surface, resulting in an increase of I_Na_ and I_NaL_ and consequent prolongation of AP duration and QT interval. The observed lack of electrophysiological phenotype in *Scn5a*-p.Y1981N mice however questions the in vivo relevance of the PY-motif for Na_v_1.5 function, at least under basal conditions.

### 3.3. Lack of In Vivo Phenotype in Scn5a-p.Y1981N Mice: Potential Underlying Mechanisms

While the *SCN5A*-p.Y1977N mutation abolished the Nedd4-2 dependent decrease in I_Na_ in HEK293 cells (in line with the predicted Na_v_1.5 gain-of-function linked to the LQT3 phenotype), mice carrying the homologous *Scn5a*-p.Y1981N mutation did not show any electrophysiological alterations. In vivo surface ECG as well as ex vivo epicardial mapping analyses in isolated hearts showed comparable ventricular conduction and repolarization indices between wild-type and *Scn5a*-p.Y1981N mice. Similarly, patch-clamp analysis conducted in isolated ventricular cardiomyocytes revealed unaltered peak I_Na_, I_NaL_ and AP properties in *Scn5a*-p.Y1981N mice. While we cannot rule out potential species differences between human and mouse, a number of plausible explanations exist for the observed lack of phenotype in *Scn5a*-p.Y1981N mice. Although it is known that the PY-motif plays a key role in channel ubiquitylation, the WW domain of Nedd4-2 can also bind to alternative sequences, including phosphorylated Px(pS/T)P, PR, and PPLP motifs [25,26,27]. Indeed, the effect of Nedd4-2 on proteins other than sodium channels, such as Kv7.3, is not abrogated upon mutation of the PY-motif [28]. Furthermore, Nedd4-2 can still downregulate Kv1.3 despite the fact that this channel does not contain a PY-motif [29]. These observations provide further evidence that Nedd4-2 has the ability to bind to sequences other than the PY-motif, providing potential alternative or compensatory ubiquitylation “reserve” mechanisms. In addition, Nedd4-2 may also indirectly modulate ion channel function through accessory proteins, which do have a PY-motif [30,31]. Indeed, in our study, the Nedd4-2 dependent decrease in WT peak I_Na_ occurred in a less pronounced manner when increasing ratios of *SCN1B* were co-transfected with *SCN5A* WT in HEK293 cells. It is therefore tempting to speculate that intrinsic differences in Na_v_1.5 interacting proteins or accessory subunits between HEK293 cells and cardiomyocytes explain (part of) the apparent discrepancies observed in our current study. Nedd4-2 function is furthermore regulated by cytokines, deubiquitinase Usp2-45, Ndfip adaptor proteins, 14-3-3 proteins and post-translational modifications such as phosphorylation [6,14,32], and it is as yet unclear if and how these differ between model and/or species (mouse versus human). Finally, other E3 ubiquitin ligases of the Nedd4 family (e.g., WWP2, SMURF) are also expressed in cardiomyocytes (RNA-Seq GEO Dataset GSE102772 [33]) and may compete with each other [10,14]. Hence, multiple mechanisms may underlie the observed differences between our observations in HEK293 cells versus *Scn5a*-p.Y1981N mice on one hand, and between our LQT3 patient and the mice on the other hand. In the patient carrying the *SCN5A*-p.Y1977N mutation, a number of (unknown) factors impacting on these mechanisms may have affected Nedd4-2 dependent modulation of Na_v_1.5, decreasing ubiquitylation reserve and predisposing to enhanced I_NaL_ and consequent QT-prolongation. Nevertheless, we cannot exclude the possibility that other genetic variants may have also contributed to the clinical phenotype.

## 4. Materials and Methods

### 4.1. Ethical Statements

Human studies were approved by the Columbia University institutional review board (approval number AAAA5720, date of approval 30 September 2003) and conformed to the principles outlined in the Declaration of Helsinki; informed consent for genetic testing was obtained from the parent. A panel of five genes for long QT (*KCNQ1, KCNH2, SCN5A, KCNE1, KCNE2*) were assessed by Sanger sequencing as previously described [13]. Animal studies conformed to the guidelines from Directive 2010/63/EU of the European Parliament on the protection of animals used for scientific purposes and were approved by the animal experiments committee of the Amsterdam UMC, Amsterdam, The Netherlands (approval number DCA318, date of approval 12 July 2018).

### 4.2. Cell Culture and Molecular Analyses

#### 4.2.1. Cell Culture

The HEK293 cell line has been purchased from A.T.C.C (ATCC-CRL-1573, Manassas, VA, USA). HEK293 cells were cultured with DMEM medium supplemented with Glutamine 4 mM, FBS 10% and gentamycine 20 μg/mL. All cell medium components except glutamine (Sigma Aldrich, Buchs, Switzerland) were purchased from Life Technologies Inc. (Life Technologies, Basel, Switzerland). HEK293 cells were maintained at 37 °C in a 5%/95% CO_2_/O_2_ incubator. HEK293 cells were transiently transfected with *SCN5A* WT (300 µg) alone or together with *SCN1B* (in a ratio of 1:1 or 1:5) with or without Nedd4-2 (1400 µg) and with (300 µg) *SCN5A*-p.Y1977N (YN) with or without Nedd4-2 (1400 µg). Transfected cells were subsequently used for molecular and electrophysiological investigation. Transfections were done using JetPEI reagent from Polyplus-Transfection (Polyplus, Illkirch, France), according to the manufacturer’s instructions.

#### 4.2.2. Quantitative RT-PCR

Total RNA was isolated from HEK293 cell pellets using TRIzol^®^ Reagent (Invitrogen,Carlsbad, CA, USA) following the manufacturer’s instructions. Reverse transcription was performed with 2 µg RNA and the High-Capacity cDNA Reverse Transcription Kit (Invitrogen, Carlsbad, CA, USA). Diluted cDNA (1:10) was used for qPCR with the TaqManTM Fast Universal PCR Master Mix (Thermo Fisher Scientific, Basel, Switzerland). qPCR conditions were as follows: 2 min activation at 50 °C, then 1 min at 95 °C; followed by 40 cycles of 3 s denaturation at 95 °C and 20 s annealing at 60 °C. Expression levels of target genes were normalized to expression of the reference gene *GAPDH* (Hs99999905_m1). The detected target sequences were *SCN5A* (Hs00165693_m1), *SCN1B* (Hs00962350_m1), and *NEDD4L* (Hs00969321_m1).

#### 4.2.3. Western Blots

HEK293 cells or homogenized whole mouse heart were lysed in 1.0 mL of lysis buffer (50 mM Hepes pH 7.4, 150 mM NaCl, 10% glycerol, 1% triton, mM EGTA supplemented with 10 mM N-ethyl maleimide and protease inhibitors (Roche, Basel, Switzerland). Protein concentrations were determined by performing Bradford assays (Coo protein dosage kit; Interchim, Montluçon, France). Forty μg of protein was loaded onto a SDS-PAGE gel. Protein transfer was performed with the dry system transfer i-blot from Invitrogen (Invitrogen, Basel, Switzerland), and immunoblotting using the snap-id system of Millipore (Millipore, Zug, Switzerland). Detection was achieved using the LICOR system^®^, and the intensity of the bands was quantified with Odyssey software (LICOR, Lincoln, NE, USA).

#### 4.2.4. Pull-Down Assays.

S5A and *SCN5A* WT, *SCN5A*-p.Y1977N (YN) or *SCN5A*-p.Y1977A (YA) C-terminus cDNAs were cloned into pGEX-4T1 (Amersham Biosciences, Otelfingen, Switzerland). Expression of GST-fusion proteins in *E. coli* bacteria was induced with 0.2 mM Isopropyl β-D-1-thiogalactopyranoside for 2 h at 29 °C. Cells were harvested by centrifugation and resuspended in lysis buffer (200 mM Tris pH 7.5, 250 mM NaCl, 1 mM EDTA, 0.5% Igepal). Following 15 min centrifugation at 13,000× *g* (4 °C), supernatants were incubated for 1 h in the presence of GSH-Sepharose beads at 4 °C. Beads were then washed three times with lysis buffer and used in pull-down experiments. Two mg total protein was added to 100 μg of GST-Na_v_1.5 C-terminus beads and incubated for 2 h at 4 °C. After washing the beads three times with lysis buffer, precipitated proteins were eluted with sampling buffer (Invitrogen, Basel, Switzerland) and analyzed by western blot.

#### 4.2.5. Cell Surface Biotinylation Assay

HEK293 cells were transiently transfected with *SCN5A* WT or YN cDNAs 48 h prior to treatment with EZ-LinkTM Sulfo-NHS-SSBiotin (Pierce, Rockford, IL, USA) at 0.35 mg/mL in cold PBS for 30 min at 4 °C. Following cell surface biotinylation, HEK293 cells were washed three times with cold Glycine solution 200 mM in PBS, plus once in cold PBS to inactivate and remove excess biotin. The cells were lysed for 45 min in lysis buffer containing (in mM): 50 HEPES pH 7.4, 125 NaCl, 1.5 MgCl_2_, 10 EGTA pH 8, 1; and 8.7% glycerol; 1% Triton X-100; 24 mg N ethylmaleimide and 1 tablet of Complete^®^ protease inhibitors cocktail. Cell lysates were spun at 16,000× *g* for 15 min, and the supernatants were incubated for 2 h at 4 °C with Steptavidin Sepharose^TM^ beads (GE Healthcare, Uppsala, Sweden). Beads were subsequently spun and washed 5 times with lysis buffer supplemented with 1% of PMSF 100 mM in isopropanol. Pelleted beads were then resuspended in 2.5 × SDS-PAGE loading buffer, denatured 30 min at 37 °C, and analyzed in 7% polyacrylamide gel electrophoresis.

#### 4.2.6. Antibodies

The anti-Na_v_1.5 polyclonal antibody ASC005 (Alomone, Jerusalem, Israel) was used at a dilution of 1/500. The monoclonal anti-actin antibody A7811 was purchased from Sigma-Aldrich (Sigma-Aldrich Chemie, Buchs, Switzerland) and used at a dilution of 1/2000. The monoclonal anti-ubiquitin FK2 (BML-PW8810-0500; Biomol, Hamburg, Germany) was used at a dilution of 1/500. A-27 polyclonal antibody against Nedd4 enzymes was a generous gift of Prof. O. Staub (University of Lausanne, Lausanne, Switzerland) and used at a dilution of 1/500.

### 4.3. Generation of Homozygous Scn5a-p.Y1981N (YN) Mice

Heterozygous *Scn5a*-p.Y1981N mice were generated utilizing Cre/loxP-mediated gene targeting. The vectors and strategy used to generate these mice were identical to those used by Remme et al. [34], except that the *Scn5a*-1798insD mutation was reverted and the p.Y1981N mutation added. A targeting construct containing a 5.5-kb SacI-SacI 5′ fragment (intron 24-intron 27 of *SCN5A*), a floxed neo cassette as a selectable marker, a 7-kb SacI-BamHI 3′ fragment (intron 28–3′UTR of *SCN5A*; the tyrosine 1981 was mutated in asparagine and a silent *Eco*RV site was introduced within the 3′ UTR), and a thymidine kinase gene for negative selection against random integration was used. Homologous recombination was carried out in 129P2/Ola Hsd embryonic stem cells. *Scn5a*-p.Y1981N stem cells were subsequently injected into C57BL/6J blastocysts to generate chimeras. To remove the neo cassette, and obtain F1 heterozygous offspring, male chimeras were crossed with Nestin-Cre mice. F1 mice were subsequently backcrossed to C57BL/6J mice (≥6 generations) establishing a line in this genetic background. Heterozygous *Scn5a*-p.Y1981N mice were crossed together to obtain homozygous *Scn5a*-p.Y1981N (YN) mice. Wild-type mice obtained during these crossings were used as a control. Both heterozygote and homozygote *Scn5a*-p.Y1981N mice were born at a Mendelian frequency. Mice were genotyped using the silent *Eco*RV site introduced within the 3′ UTR as previously described [34]. All experiments were performed on adult (3–6-months old) male homozygous (YN) and wild-type mice, and were in accordance with governmental and institutional guidelines for animal use in research.

### 4.4. Surface ECG Analysis

Mice were anesthetized using isoflurane inhalation (0.8–1.0 volume % in oxygen) and surface ECGs were recorded from subcutaneous 23-gauge needle electrodes attached to each limb using the Powerlab acquisition system (ADInstruments Ltd, Oxford, United Kingdom). ECG traces were signal averaged and analyzed for heart rate (RR-interval), P-wave, PR-, QRS- and QT-interval duration using the LabChart7Pro software (ADInstruments Ltd, Oxford, United Kingdom) RR interval was defined as the interval in ms between two consecutive R waves. PR-, P-wave, QRS- and QT-intervals were determined as indicated in Figure 4A. QT-intervals were corrected for heart rate using the formula: QTc = QT/(RR/100)^1/2^ (RR in ms) [35].

### 4.5. Epicardial Mapping in Isolated Hearts

Mice were sacrificed by CO_2_ administration (100% CO_2_ 20% *v*/*v* per minute) followed by cervical dislocation, after which the heart was excised, cannulated, placed in an optical mapping setup perfused at 37 °C and incubated with a modified Tyrode’s solution containing (in mM): 128 NaCl, 4.7 KCl, 1.45 CaCl_2_, 0.6 MgCl_2_, 27 NaHCO_3_, 0.4 NaH_2_PO_4_, 11 glucose, 0.015 Di-4 ANEPPS (pH maintained at 7.4 by equilibration with a mixture of 95% O_2_ and 5% CO_2_) for 15 min. Subsequently hearts were perfused with the solution described above without Di-4 ANEPPS but with the addition of blebbistatin to prevent movement artifacts. Excitation light was provided by a 5-watt power LED (filtered 510 ± 20 nm). Fluorescence (filtered > 610 nm) was transmitted through a tandem lens system on a CMOS sensor (100 × 100 elements; MiCAM Ultima, SciMedia, Costa Mesa, CA, USA) Hearts were paced at basic cycle length of 120 ms or 150 ms at twice the diastolic stimulation threshold from the center of the ventricular epicardial surface. Optical action potentials were analyzed using custom software. Local activation was defined as the maximum positive slope of the action potential, and local activation times were used to construct ventricular activation maps. Longitudinal and transversal conduction velocities (CVs) were determined from ventricular activation maps. To calculate CVs in longitudinal and transversal directions, the difference in activation time was determined between two points separated by a known distance and located parallel (longitudinal) or perpendicular (transversal) to the direction of propagation. The direction of propagation was determined using isochronal maps. Three parallel values of CV in each of the two directions were acquired and averaged. Repolarization maps were constructed using APD values at 50% (APD_50_) and 80% of repolarization (APD_80_). For each heart, average values of APD_50_ and APD_80_ were calculated from five randomly chosen locations on the left ventricular epicardial surface.

### 4.6. Patch-Clamp Measurements

#### 4.6.1. Isolation of Left Ventricular Cardiomyocytes

Mice were sacrificed by CO_2_ administration (100% CO_2_ 20% *v*/*v* per minute) followed by cervical dislocation, after which the heart was excised, cannulated, mounted on a Langendorff set-up, and perfused at 37 °C for 8 min with normal Tyrode’s solution containing (in mM): 140 NaCl, 5.4 KCl, 1.8 CaCl_2_, 1.0 MgCl_2_, 5.5 glucose, 5 HEPES; pH 7.4 (NaOH). Next, the heart was perfused for 8 min with a similar solution in which the calcium concentration was lowered to 1.08 × 10^−5^ M (low- calcium solution), and the enzyme Liberase Blendzyme type 4 (Roche Diagnostics, GmbH, Mannheim, Germany) was added at a concentration of 0.055 mg/mL. Then, digested tissue was gently triturated in the low-calcium solution and single cardiomyocytes were washed twice in the low-calcium solution supplemented with BSA (1 mg/mL), and twice in normal Tyrode’s solution at 37 °C. Isolated ventricular cardiomyocytes were stored at room temperature and used within 4 h.

#### 4.6.2. Data Acquisition and Analysis

Peak I_Na_, I_NaL_ and action potentials (APs) were measured with the ruptured and perforated patch-clamp technique, respectively, using an Axopatch 200B amplifier (Molecular Devices, San Jose, CA, USA) for the recordings in cardiomyocytes and HEK293 cells. Voltage control, data acquisition, analysis of I_Na_, I_NaL_ and APs were performed with pClamp10.2/Clampfit (Molecular Devices, San Jose, CA, USA) and custom-made software. Borosilicate glass patch pipettes with a tip resistance of 2–2.5 MΩ were used. Cell membrane capacitance (Cm) was determined by dividing the decay time constant of the capacitive transient in response to 5 mV hyperpolarizing steps from −40 mV, by the series resistance. Series resistance and cell membrane capacitance were compensated for ≥80%. Peak I_Na_ and APs were filtered at 5 kHz and digitized at 40 kHz, while I_NaL_ was filtered and digitized at 2 kHz and 1 kHz, respectively.

#### 4.6.3. Sodium Current Measurements

In isolated cardiomyocytes, peak I_Na_ and I_NaL_ were measured using a pipette solution containing (in mM): 3.0 NaCl, 133 CsCl, 2.0 MgCl_2_, 2.0 Na_2_ATP, 2.0 TEACl, 10 EGTA, 5.0 HEPES; pH 7.2 (CsOH). Bath solution for I_NaL_ measurements contained: 130 NaCl, 10 CsCl, 1.8 CaCl_2_, 1.2 MgCl_2_, 11.0 glucose, 5.0 HEPES; 0.005 nifedipine; pH 7.4 (CsOH). Bath solution composition for peak I_Na_ recordings was similar to the I_NaL_ bath solution with the exception of a lower NaCl concentration for proper voltage control. Hence, the peak I_Na_ bath solution contained (in mM): 7 NaCl, 133 CsCl, 1.8 CaCl_2_, 1.2 MgCl_2_, 11.0 glucose, 5.0 HEPES; 0.005 nifedipine; pH 7.4 (CsOH). For peak I_Na_ measurements in HEK293 cells the pipette solution contained (in mM): 60 CsCl, 50 aspartic acid, 1 CaCl_2_, 1 MgCl_2_, 10 HEPES, 5 Na_2_ATP, 11 EGTA; pH 7.2 (CsOH). The bath solution contained (in mM): 25 NaCl, 105 NMDG-Cl, 2 CaCl_2_, 1.2 MgCl_2_, 5 CsCl, 10 HEPES, 20 glucose; pH 7.4 (HCl). I_NaL_ was measured at 36 °C, as tetrodotoxin (TTX)-sensitive current, using a descending ramp protocol from a holding potential of –90 mV at a cycle length of 5 s. Peak I_Na_ was measured at room temperature in both HEK293 and cardiomyocytes. In cardiomyocytes, I_Na_ was elicited from a holding potential of –120 mV with a cycle length of 5 s, while in HEK293 cells the holding potential was –100 mV and the cycle length 3 s. I_Na_ was defined as the difference between peak and steady state current at the end of the step. Current densities were calculated by dividing current amplitude by Cm. Potentials were not corrected for the estimated change in liquid junction potential. Voltage dependence of activation and inactivation curves were fitted with Boltzmann function (y = [1 + exp{(*V* − *V*_1/2_)/*k*}]^−1^), where *V*_1/2_ is the half-maximal voltage of (in)activation and *k*, the slope factor. Recovery from inactivation was assessed using a double pulse protocol. Data were normalized to the values of the current obtained during the first pulse (P_1_) and fitted with a bi-exponential function (y = y_0_ + *A_f_*{1 − exp[−t/τ_f_]} + *A_s_*{1 − exp[−t/τ_s_]}), where *A_f_* and *A_s_* represent the amplitudes of the fast and the slow components of recovery from inactivation, and τ_f_ and τ_s_ their respective recovery time constants.

#### 4.6.4. Action Potential Measurements

In single left ventricular cardiomyocytes, APs were measured at 36 °C using a normal Tyrode’s solution containing (in mM): 140 NaCl, 5.4 KCl, 1.8 CaCl_2_, 1.0 MgCl_2_, 5.5 glucose, 5 HEPES, pH 7.4 (NaOH). Pipettes were filled with (in mM): 125 K-gluconate, 20 KCl, 5 NaCl, 0.22 amphotericin-B, 10 HEPES, pH 7.2 (KOH). APs were elicited at the stimulation frequency of 2 Hz and 4 Hz by 3 ms, ≈1.2 × threshold current pulses through the patch pipette. AP recordings were initiated only when access resistance (R_a_), resting membrane potential (RMP), and AP duration were stable. AP measurements presenting R_a_ > 30 MΩ were not included in the manuscript. Proper R_a_ values were generally obtained 10 min after the formation of the seal. The maximal AP upstroke velocity (V_max_) was determined from the first derivative (dV/dt) of the AP upstroke, with the maximal dV/dt denoting the V_max_. Moreover, we analyzed RMP, AP amplitude, APD_20_, APD_50_ and APD_90_. Data from 10 consecutive APs were averaged and potentials were corrected for the calculated liquid junction potential (15 mV).

### 4.7. Statistical Analysis

Values are shown as mean ± SEM. Unpaired Student’s t-test, two-way repeated measures ANOVA followed by Holm-Sidak test for post-hoc analysis and one-way ANOVA followed by Holm-Sidak test for post-hoc analysis were used when appropriate. The Mann-Whitney U test and Kruskal-Wallis One Way ANOVA were used for data not normally distributed. The level of statistical significance was set to *p* < 0.05.

## 5. Conclusions

We have shown that the LQT3-associated *SCN5A* mutation p.Y1977N located in the PY-motif of Na_v_1.5 disrupts Nedd4-2 binding and consequent ubiquitylation in vitro. Nevertheless, mice carrying the homologous *Scn5a*-p.Y1981N mutation showed no electrical disturbances nor alterations in (late) I_Na_ and AP properties. These findings suggest the presence of compensatory mechanisms, with additional, as yet unknown, factors likely required to reduce the “ubiquitylation reserve” of Na_v_1.5 and unmask alterations in *Scn5a*-p.Y1981N channel degradation and (gain-of-) function. Future identification of such modulatory factors may identify potential triggers for arrhythmias and sudden cardiac death in the setting of LQT3 mutations. Importantly, discrepancies between the in vitro, in vivo and ex vivo results observed in this study underscore the importance of investigating the functional consequences of *SCN5A* mutations in cardiomyocytes and cautions against drawing conclusions from findings obtained solely in heterologous expression systems.

## Supplementary Materials

Supplementary materials can be found at https://www.mdpi.com/1422-0067/20/20/5033/s1. Figure S1: Raw images of the Western blot of Figure 1B; Figure S2: Raw images of the Western blot of Figure 1C; Figure S3: Raw image of the Western blot of Figure 1D; Figure S4: Raw images of the Western blot of Figure 3A; Figure S5: Raw images of the Western blot of Figure 3C; Figure S6: Nedd4-2-dependent regulation of wild-type sodium current in the presence of increasing ratios of the accessory subunit *SCN1B*; Figure S7: *SCN5A*, *SCN1B* and *NEDD4L* (Nedd4-2) transcript levels in HEK293 cells transfected with increasing ratios of the accessory subunit *SCN1B*; Figure S8: Action potential properties in wild-type and *Scn5a*-p.Y1981N cardiomyocytes at 4 Hz; Table S1: Sodium current biophysical properties in HEK293 cells transiently transfected with *SCN5A* wild-type (WT) or *SCN5A*-p.Y1977N (YN), with or without Nedd4-*2*; Table S2: Sodium current biophysical properties in HEK293 cells transiently transfected with *SCN5A* wild-type (WT) and *SCN1B* (β_1_-subunit) at a ratio of 1:1 or 1:5 with or without Nedd4-*2*; Table S3: Action potential characteristics at the stimulation frequency of 2 Hz and 4Hz for left ventricular cardiomyocytes isolated from wild-type (WT) and *Scn5a*-p.Y1981N (YN) mice; Table S4: Sodium current biophysical properties in left ventricular cardiomyocytes isolated from wild-type (WT) and *Scn5a*-p.Y1981N (YN) mice.

## Abbreviations

AP	Action potential
APD	Action potential duration
APD_20_	Action potential duration at 20% repolarization
APD_50_	Action potential duration at 50% repolarization
APD_80_	Action potential duration at 80% repolarization
APD_90_	Action potential duration at 90% repolarization
APA	Action potential amplitude
V_max_	Maximal upstroke velocity
RMP	Resting membrane potential
LQT3	Long QT syndrome type 3
I_NaL_	Late sodium current
I_Na_	Sodium current
BrS	Brugada syndrome
CCD	Cardiac conduction disease
SCD	Sudden cardiac death
ICD	Implantable cardioverter defibrillator
WT	Wild-type
TTX	Tetrodotoxin
hiPSC-CMs	Human induced pluripotent stem cell-derived cardiomyocytes
V_1/2_	Half-maximal voltage of (in)activation
CVs	Conduction velocities
τ_f_	Fast time constant of recovery from inactivation
τ_s_	Slow time constant of recovery from inactivation
