# Supplementary material for: Functional Consequences of the SCN5A-p.Y1977N Mutation within the PY Ubiquitylation Motif: Discrepancy between HEK293 Cells and Transgenic Mice
Source: Int J Mol Sci. 2019 Oct 11;20(20):5033. doi: 10.3390/ijms20205033 (PMC6829230; doi:10.3390/ijms20205033)
Supplement: Supplementary file 1 [file ijms-20-05033-s001.pdf]

Supplementary Materials

Functional consequences of the *SCN5A*-p.Y1977N mutation within the PY ubiquitylation motif: discrepancy between HEK293 cells and transgenic mice

Simona Casini*, Maxime Albesa*, Zizun Wang, Vincent Portero, Daniela Ross-Kaschitza, Jean-Sébastien Rougier, Gerard A. Marchal, Wendy K. Chung, Connie R. Bezzina, Hugues Abriel*, Carol Ann Remme*

* These authors contributed equally

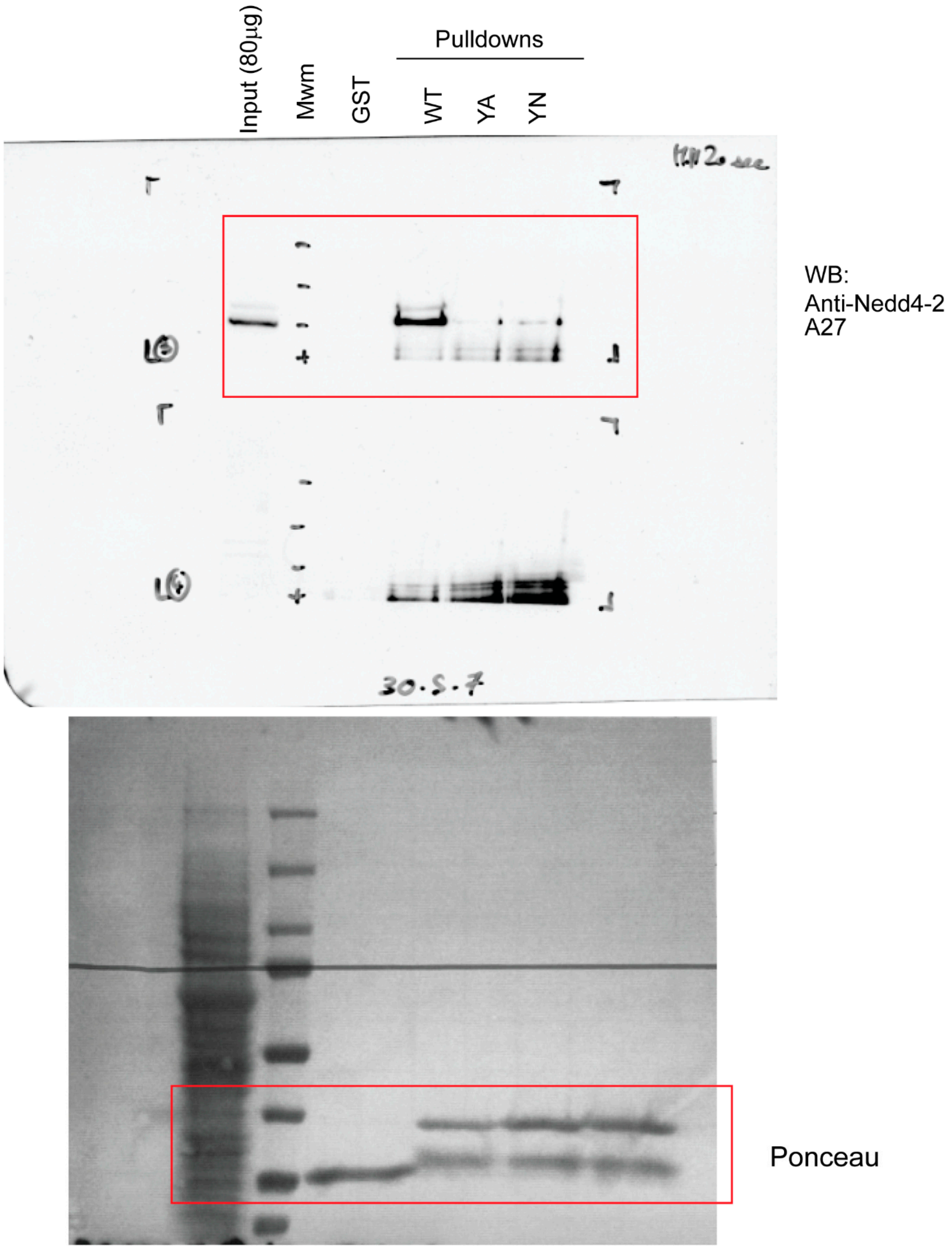

Figure S1. **Raw images of the Western blot of Figure 1B**. Uncropped version of the Western blot reported in Figure 1B of the manuscript. Red rectangles indicate the areas shown in Figure 1B.

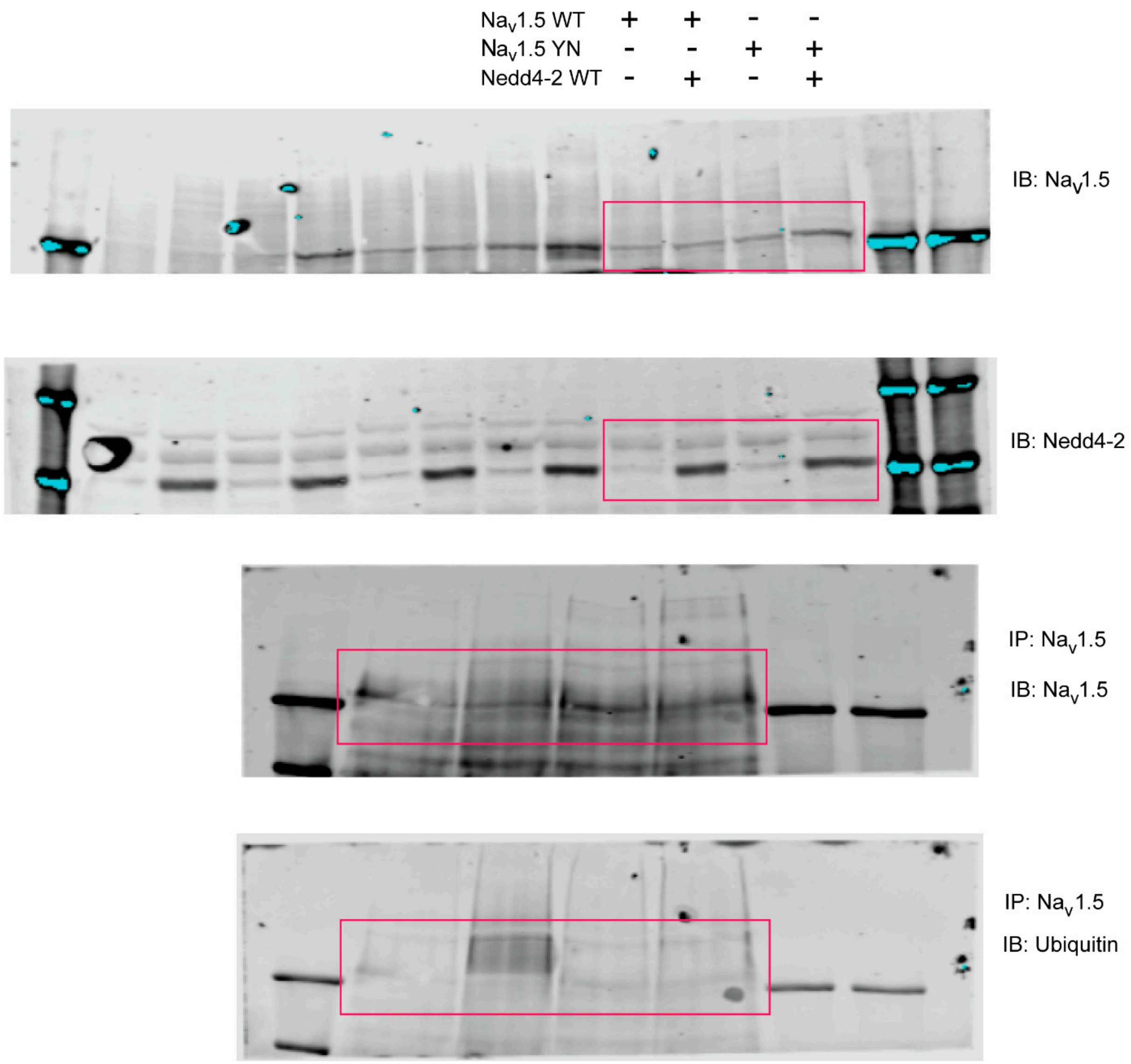

Figure S2. **Raw images of the Western blot of Figure 1C**. Uncropped version of the Western blot reported in Figure 1C of the manuscript. Red rectangles indicate the areas shown in Figure 1C.

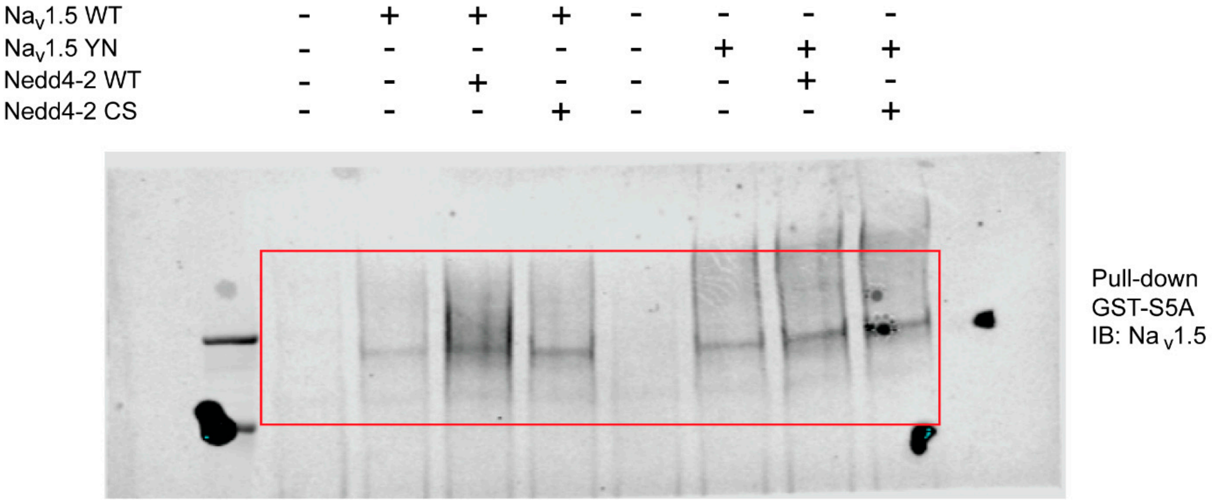

Figure S3. **Raw image of the Western blot of Figure 1D**. Uncropped version of the Western blot presented in Figure 1D of the manuscript. Red rectangle indicates the area shown in Figure 1D.

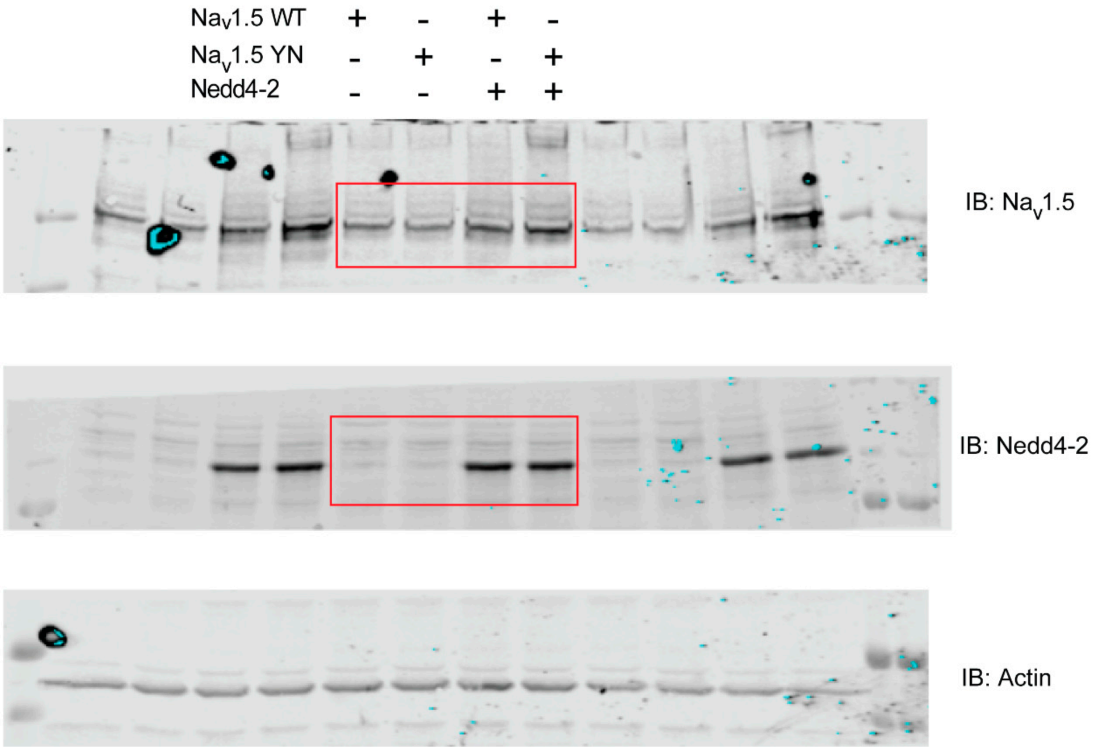

Figure S4. Raw images of the Western blot of Figure 3A. Uncropped version of the Western blot presented in Figure 3A of the manuscript. Red rectangles indicate the areas shown in Figure 3A.

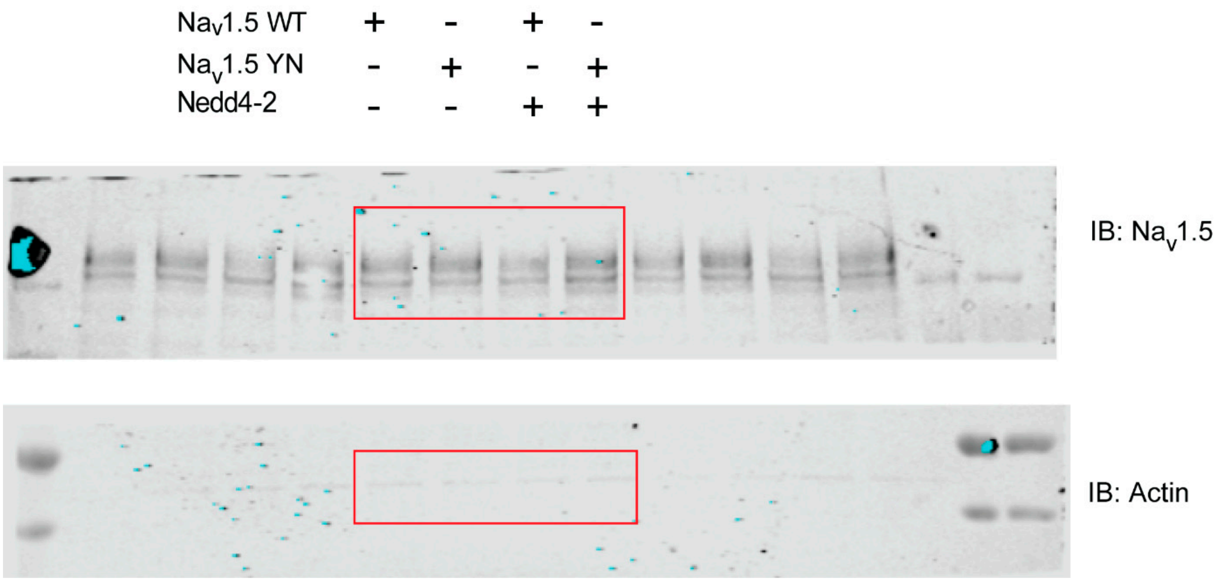

Figure S5. Raw images of the Western blot of Figure 3C. Uncropped version of the Western blot presented in Figure 3C of the manuscript. Red rectangles indicate the areas shown in Figure 3C.

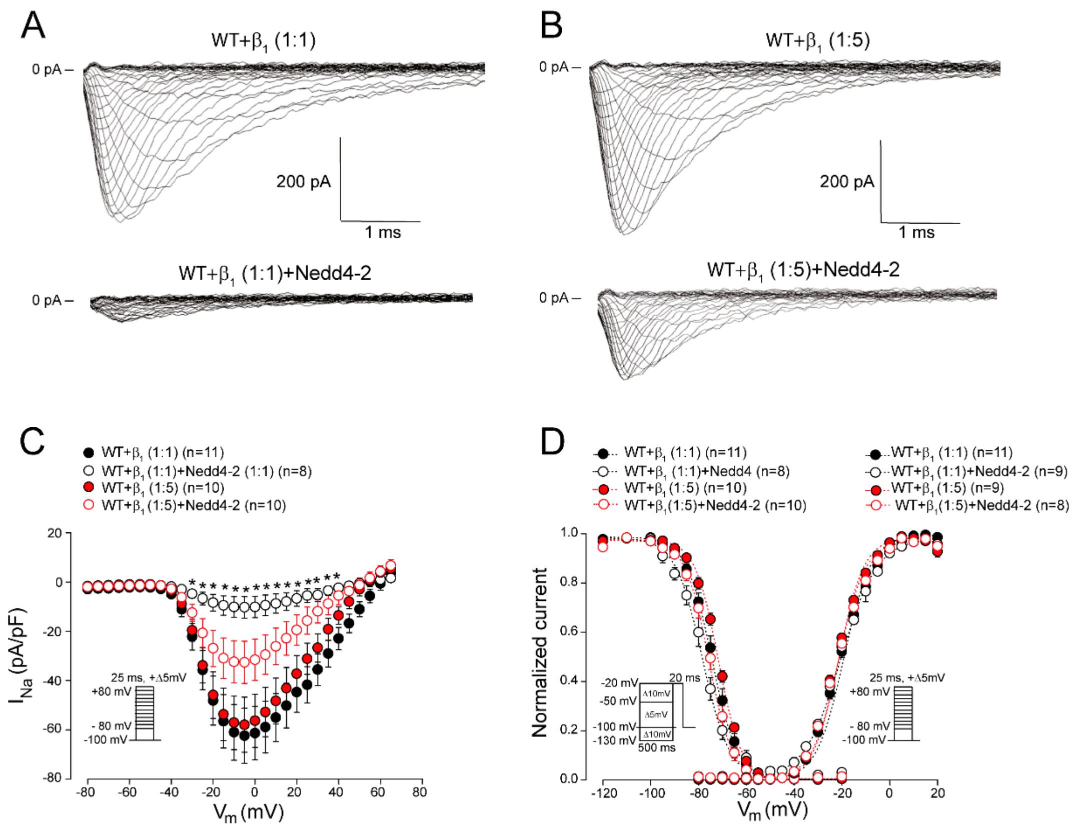

Figure S6. **Nedd4-2-dependent regulation of wild-type sodium current in the presence of increasing ratios of the accessory subunit *SCN1B*.** Typical example of sodium current recordings obtained in HEK293 cells transiently transfected with wild-type *SCN5A* (WT) and *SCN1B* (β_1-subunit) at a ratio of 1:1 (**A**) or 1:5 (**B**) with or without Nedd4-2. Average sodium current-voltage relationship **(C)** and voltage dependence of activation and inactivation **(D)** for HEK293 cells transiently transfected with WT and β_1-subunit at a ratio of 1:1 or 1:5 with or without Nedd4-2. Insets: voltage clamp protocols. *$p<0.05$ *vs* WT+β_1 (1:1) two-way repeated measures ANOVA followed by Holm-Sidak test for post-hoc analysis. n indicates the number of cells.

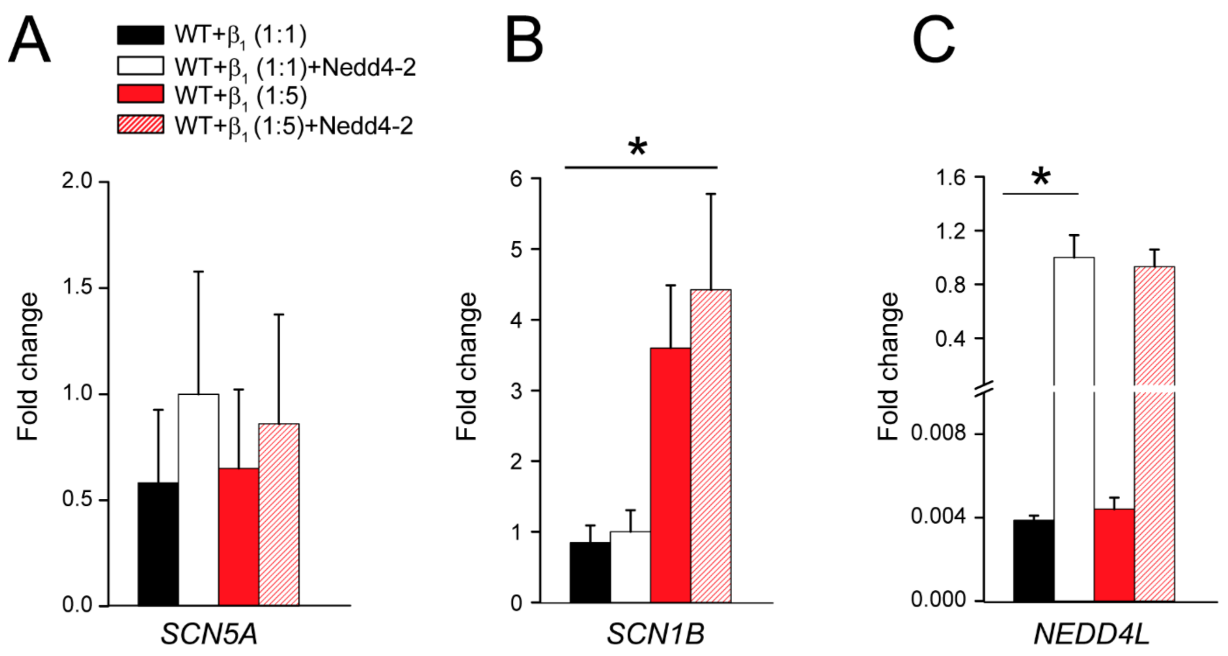

Figure S7. ***SCN5A*, *SCN1B* and *NEDD4L* (Nedd4-2) transcript levels in HEK293 cells transfected with increasing ratios of the accessory subunit *SCN1B*.** Expression levels for *SCN5A* (A), *SCN1B* (B) and *NEDD4L* (Nedd4-2) (C) in HEK293 cells transiently transfected with wild-type *SCN5A* (WT) and *SCN1B* (β_1-subunit) at a ratio of 1:1 or 1:5 with or without Nedd4-2. Data are expressed as fold change compared to the WT+β_1(1:1)+Nedd4-2 group and normalized for *GAPDH.* Depicted data are average and SEM of 4 independent biological replicates.*$p<0.05$ Kruskal-Wallis one way ANOVA followed by Tukey test for post-hoc analysis.

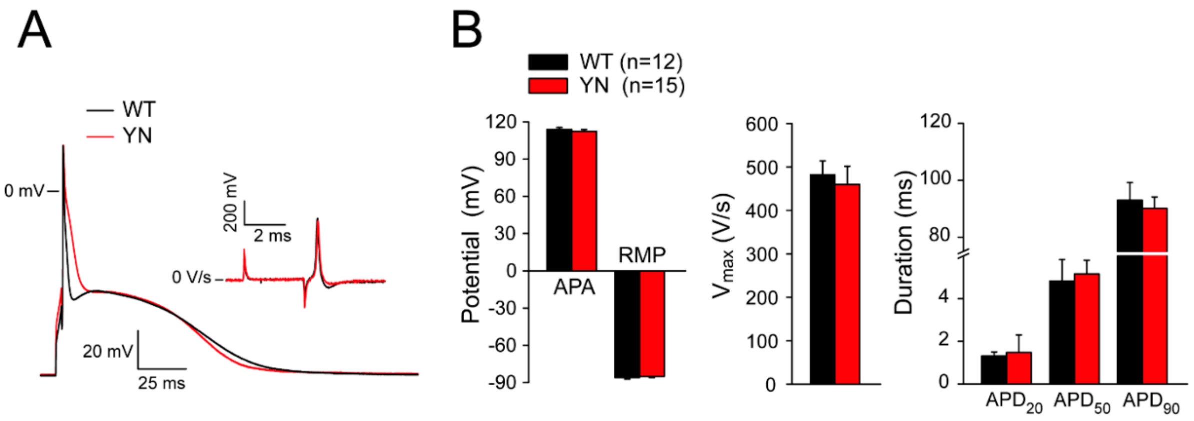

Figure S8. Action potential properties in wild-type and *Scn5a*-p.Y1981N cardiomyocytes at 4 Hz. (A) Typical examples of action potentials (APs) triggered at 4 Hz and first derivative (dV/dt) of the AP upstroke (inset) in wild-type (WT) and *Scn5a*-p.Y1981N (YN) mouse cardiomyocytes. **(B)** Average values for AP amplitude (APA), resting membrane potential (RMP), maximal upstroke velocity (V_{max}; measured as the maximal dV/dt of the AP upstroke) and AP duration at 20%, 50% and 90% repolarization (APD_{20}, APD_{50}, APD_{90}) at the stimulation frequency of 4 Hz in WT (n=12 cardiomyocytes from 5 mice) and YN cardiomyocytes (n=15 from 5 mice).

Table S1: Sodium current biophysical properties in HEK293 cells transiently transfected with *SCN5A* wild-type (WT) or *SCN5A*-p.Y1977N (YN), with or without Nedd4-2

	**WT**	**n**	**WT+Nedd4-2**	**n**	**YN**	**n**	**YN+Nedd4-2**	**n**
Current density								
**I_{Na} (pA/pF)**	-69.2±13.9	10	-23.4±8.0$^{*,\#}$	8	-66.5±9.4	11	-67.0±9.5	11
Activation								
**$V_{1/2}$ (mV)**	-22.2±1.3	10	-19.8±1.6	8	-21.8±0.9	11	-22.4±0.8	11
***k* (mV)**	6.7±0.2	10	6.5±0.3	8	6.9±0.3	11	6.7±0.2	11
Inactivation								
**$V_{1/2}$ (mV)**	-75.1±1.0	11	-74.8±0.7	7	-74.9±0.8	13	-77.4±0.8	8
***k* (mV)**	-4.8±0.2	11	-4.6±0.2	7	-5.0±0.2	13	-5.6±0.2$^{\$}$	8

I_{Na}, sodium current density measured at -5 mV; $V_{1/2}$ of (in)activation, half-voltage of (in)activation; *k,* slope of the (in)activation curves; *$p<0.05$ *vs* WT, $^{\#}p<0.05$ vs YN+Nedd4-2 (two-way repeated measures ANOVA followed by Holm-Sidak test for post-hoc analysis, as indicated in Figure 2); $^{\$}p<0.05$ *vs* WT+Nedd4-2, one way ANOVA followed by Holm-Sidak test for post-hoc analysis.

Table S2. Sodium current biophysical properties in HEK293 cells transiently transfected with *SCN5A* wild-type (WT) and *SCN1B* (β_1-subunit) at a ratio of 1:1 or 1:5 with or without Nedd4-2

	**WT+β_1 (1:1)**	**n**	**WT+β_1 (1:1) +Nedd4-2**	**n**	**WT+β_1 (1:5)**	**n**	**WT+β_1 (1:5) +Nedd4-2**	**n**
Current density								
**I_{Na} (pA/pF)**	-62.4±11.2	11	-10.2±4.4*	8	-58.0±11.2	10	-32.6 ±8.6	10
Activation								
**$V_{1/2}$ (mV)**	-20.0±0.7	11	-20.1±0.8	8	-21.7±0.6	10	-20.9±1.0	10
***k* (mV)**	7.0±0.2	11	9.0±0.6$^{\$}$	8	6.5±0.1	10	7.1±0.3	10
Inactivation								
**$V_{1/2}$ (mV)**	-74.3±1.1	11	-78.4±1.1$^{\#,\&}$	9	-72.1±0.5	10	-75.6±1.1	8
***k* (mV)**	-5.4±0.2	11	-7.0±0.7	9	-5.4±0.3	10	-5.3±0.3	8

I_{Na}, sodium current density measured at -5 mV; $V_{1/2}$ of (in)activation, half-voltage of (in)activation; *k*, slope of the (in)activation curves; *p<0.05 *vs* WT+β_1 (1:1), two-way repeated measures ANOVA followed by Holm-Sidak test for post-hoc analysis, as indicated inFigure S6; . $^{\$}$p<0.05 *vs* WT+$\beta_1$ (1:5), $^{\#}$p<0.05 *vs* WT+β_1 (1:1), $^{\&}$p<0.05 *vs* WT+β_1 (1:5); one way ANOVA followed by Holm-Sidak test for post-hoc analysis or Kruskal-Wallis one way ANOVA followed by Dunn's test for post-hoc analysis when data were not normally distributed.

Table S3: Action potential characteristics at the stimulation frequency of 2 Hz and 4Hz for left ventricular cardiomyocytes isolated from wild-type (WT) and *Scn5a*-p.Y1981N (YN) mice.

	**WT (2Hz) (n=10)**	**YN (2Hz) (n=14)**	**WT (4Hz) (n=12)**	**YN (4Hz) (n=15)**
**RMP (mV)**	-84.7±0.7	-84.2±0.7	-85.9±0.5	-85.1±0.9
**APA (mV)**	113.9±1.7	112.6±1.5	113.9±1.6	112.4±1.5
**V_{max} (V/s)**	516.3±46.3	485.3±42.7	482.1±31.8	459.8±42.0
**APD_{20}**	1.2±0.2	1.3±0.2	1.3±0.2	1.5±0.2
**APD_{50}**	4.0±0.8	4.7±0.6	4.8±1.0	5.2±0.6
**APD_{90}**	105.4±5.3	98.1±3.6	93.0±6.3	90.1±4.0

RMP, resting membrane potential; APA, action potential amplitude; V_{max}, maximal upstroke velocity; APD_{20}, APD_{50}, APD_{90}, action potential duration at 20%, 50%, 90% repolarization.

Table S4: Sodium current biophysical properties in left ventricular cardiomyocytes isolated from wild-type (WT) and *Scn5a*-p.Y1981N (YN) mice

	WT	n	YN	n
Current density				
**I_{Na} (pA/pF)**	-56.0±2.7	8	-51.4±4.3	7
Activation				
**$V_{1/2}$ (mV)**	-44.8±0.7	8	-42.2 ±1.4	7
**k (mV)**	4.8 ±0.2	8	5.1±0.3	7
Inactivation				
**$V_{1/2}$ (mV)**	-81.4±1.2	8	-80.3±1.7	7
**k (mV)**	-5.0±0.3	8	-5.7±0.2	7
Recovery from inactivation				
**τ_{fast} (ms)**	3.9±0.2	6	5.0 ±0.6	6
**τ_{slow} (ms)**	38.0±1.8	6	44.2±4.2	6

I_{Na}, sodium current density measured at -30 mV; $V_{1/2}$ of (in)activation, half-voltage of (in)activation; *k,* slope of the (in)activation curves; $\tau_{fast,}$ fast time constant of recovery from inactivation; τ_{slow}, slow time constant of recovery from inactivation.
